# Characterization of the Metabolic Phenotype of Rapamycin-Treated CD8^+^ T Cells with Augmented Ability to Generate Long-Lasting Memory Cells

**DOI:** 10.1371/journal.pone.0020107

**Published:** 2011-05-17

**Authors:** Shan He, Koji Kato, Jiu Jiang, Daniel R. Wahl, Shin Mineishi, Erin M. Fisher, Donna M. Murasko, Gary D. Glick, Yi Zhang

**Affiliations:** 1 Department of Internal Medicine, University of Michigan, Ann Arbor, Michigan, United States of America; 2 Department of Bioscience and Biotechnology, Drexel University, Philadelphia, Pennsylvania, United States of America; 3 Chemical Biology Doctoral Program, University of Michigan, Ann Arbor, Michigan, United States of America; 4 Department of Chemistry, University of Michigan, Ann Arbor, Michigan, United States of America; University of California, San Francisco, United States of America

## Abstract

**Background:**

Cellular metabolism plays a critical role in regulating T cell responses and the development of memory T cells with long-term protections. However, the metabolic phenotype of antigen-activated T cells that are responsible for the generation of long-lived memory cells has not been characterized.

**Design and Methods:**

Using lymphocytic choriomeningitis virus (LCMV) peptide gp33-specific CD8^+^ T cells derived from T cell receptor transgenic mice, we characterized the metabolic phenotype of proliferating T cells that were activated and expanded *in vitro* in the presence or absence of rapamycin, and determined the capability of these rapamycin-treated T cells to generate long-lived memory cells *in vivo*.

**Results:**

Antigen-activated CD8^+^ T cells treated with rapamycin gave rise to 5-fold more long-lived memory T cells *in vivo* than untreated control T cells. In contrast to that control T cells only increased glycolysis, rapamycin-treated T cells upregulated both glycolysis and oxidative phosphorylation (OXPHOS). These rapamycin-treated T cells had greater ability than control T cells to survive withdrawal of either glucose or growth factors. Inhibition of OXPHOS by oligomycin significantly reduced the ability of rapamycin-treated T cells to survive growth factor withdrawal. This effect of OXPHOS inhibition was accompanied with mitochondrial hyperpolarization and elevation of reactive oxygen species that are known to be toxic to cells.

**Conclusions:**

Our findings indicate that these rapamycin-treated T cells may represent a unique cell model for identifying nutrients and signals critical to regulating metabolism in both effector and memory T cells, and for the development of new methods to improve the efficacy of adoptive T cell cancer therapy.

## Introduction

Cellular metabolism plays an important role in regulating T cell proliferation and survival. Resting T cells, including both naïve and memory T cells, derive most of their adenosine triphosphate (ATP) from basal levels of oxidative phosphorylation (OXPHOS) [1], [2], [3]. In the absence of extrinsic signals for maintaining OXPHOS, resting T cells undergo progressive atrophy [1], [2], [3]. Upon activation, T cells undergo a metabolic conversion to dramatically upregulated glycolysis that leads to the increase in production of ATP and metabolic intermediates required for cell growth and proliferation [4], [5]. Without sufficient support for their bioenergetic and biosynthetic demands, activated T cells may be deleted or become quiescent [5], [6].

Memory T cells are one of the most important components of long-term immunity against infections and tumors [7], [8], [9], [10], [11], [12], [13], [14]. They have the ability to persist within the individual for long periods and to rapidly respond to rechallenge of the antigen. As compared to naïve T cells (T_N_), memory T cells have fewer requirements of costimulating signals for their activation, proliferation and function [7], [8], [9], [10], [11], [12], [13], [14]. Thus, enhanced formation of memory T cells may improve the efficacy of vaccine and T cell therapy for the treatment of infectious diseases and cancers [13].

Memory T cells derive from antigen-activated precursor cells during primary immune responses [14], [15], [16], [17], [18]. Deletion of Traf6, a signal important for fatty acid oxidation, impaired the development of memory T cells [19], suggesting a critical role for cellular metabolism in regulating memory T cell development. However, the metabolic phenotype of proliferative memory T cell precursors has not been characterized.

Rapamycin specifically inhibits the mammalian target of rapamycin (mTOR) pathway and is an important regulator of cellular metabolism [20], [21], [22], [23], [24], [25]. For example, some studies suggested that rapamycin decreased OXPHOS [20], [21], whereas some others suggested that rapamycin enhanced the oxidation of both fatty acids and amino acids [22], [23], [24]. It appears that rapamycin may regulate OXPHOS of nutrients in a context dependent manner, possibly due to the nature of other signaling pathways activated in the target cell [20], [21], [22], [23], [24]. Interestingly, several studies demonstrate that rapamycin enhanced the survival of activated T cells [26], [27], [28], [29], [30] and generation of long-lived memory T cells [19], [31], [32]. Data from these studies indicate that rapamycin-treated T cells could represent a unique cellular model for characterizing the metabolic phenotype of antigen-activated T cells with enhanced ability to become memory T cells.

Using T cell receptor (TCR) transgenic CD8^+^ T cells that are specific for lymphocytic choriomeningitis virus (LCMV) peptide gp33, we characterized the metabolic phenotype of antigen-activated T cells that were expanded in cultures treated with or without rapamycin. We found that rapamycin-treated T cells gave rise to 5-fold more long-lived memory T cells than untreated control T cells. These rapamycin-treated T cells upregulated both glycolysis and OXPHOS and had a greater ability than control T cells to survive withdrawal of either glucose or growth factors. Inhibition of OXPHOS in rapamycin-treated T cells resulted in their elevation of reactive oxygen species (ROS), reduction of mitochondrial hyperpolarization and impaired survival capability. These observations suggest that antigen-activated T cells with increased OXPHOS may have augmented ability to survive and form memory T cells *in vivo*.

## Materials and Methods

### Mice

We purchased C57BL/6 (B6; H-2D^b^, Thy1.2^+^), Thy1.1^+^ B6 and B6/SJL (CD45.1^+^) from Jackson Laboratory (Maine, USA). C57BL/6-Tg (TCR LCMV P14)-Tcrα^tm1Mom^ (P14 B6, H-2D^b^, Thy1.2^+^, TCRα^−/−^) were obtained from Taconic (Maryland, USA). Animal work has been conducted according to relevant national and international guidelines. The Institutional Animal Care and Use Committee of the University of Michigan approved all mouse protocols (Approval ID# 09883).

### Antibodies (Abs), cell lines, cytokines and flow cytometry analysis

Abs were obtained from BD Bioscience and Biolegend. Microbead-conjugated Abs and microbead-conjugated streptavidin were purchased from Miltenyi-Biotech, and all recombinant cytokines including IL-2, IL-4, granulocyte-monocyte colony-stimulating factor, stem cell factor and tumor necrosis factor-α were from R&D Systems. We performed immunofluorescence analyses of cell surface phenotypes and intracellular cytokines using FACScan and Canto cytometer (Becton Dickinson) as previously described [33], [34].

### Cell preparation

CD8^+^ T cells were magnetically isolated from spleens and lymph nodes (LN) of mice using microbead-conjugated anti-CD8 Ab (MiniMACS, Miltenyi Biotech). To further separate CD44^lo^CD8^+^ T_N_, purified CD8^+^ T cells were stained with biotin-conjugated anti-CD44 Ab, followed by streptavidin-conjugated Dynal Magnetic beads (Invitrogen). The purity of isolated CD44^lo^CD8^+^ T_N_ was consistently more than 95%. Donor CD8^+^ T cells were labeled with fluorescent dye carboxyfluorescein diacetate succinimidyl ester (CFSE) as described [34]. Mature dendritic cells (DCs) were prepared from B6 bone marrow (BM) as previously described [35].

### *In vitro* stimulation of CD8^+^ T cells

Purified P14 CD8^+^ T_N_ were cultured in IMDM containing 10% FBS (complete medium) supplemented with IL-2 (5 ng/ml), LCMV gp33 peptide (10^−8^ M) and B6 BM DC at a T cell/DC ratio of 8∶1. In some experiments, antigen-activated CD8^+^ T cell subsets were highly purified using BD FACSAria™ III cell sorter (Becton Dickinson). The purity of these isolated P14 CD8^+^ memory T cells was consistently more than 95%.

### Real-time reverse transcription polymerase chain reaction (RT-PCR)

Total RNA was extracted from cells using TRIzol (Invitrogen Life Technologies, Carlsbad, CA). Real-time RT-PCR was performed using SYBR green PCR mix (ABI Biosystem, CA) on a Mastercycler realplex (Eppendorf AG, Westbury, NY). Transcript abundance was calculated using the ΔΔCt method (normalization with *Gapdh*). The primer sequences used for real-time RT-PCR include: GAPDH (5′-AGGTCGGTGTGAACGGATTTG and 3′-ACTGGAGTTGATGTACCAGATGT), Granzyme B (GzmB, 5′-CCACTCTCGACCCTACATGG and 3′-GGCCCCCAAAGTGACATTTATT), Bak (5′-CAGATGGATCGCACAGAGAG and 3′-TCTGTGTACCACGAATTGGC), Bax (5′-ACTAAAGTGCCCGAGCTGAT and 3′-ATGGTCACTGTCTGCCATGT), Bcl-xL (5′-GGATGGCCACCTATCTGAAT and 3′-TGTTCCCGTAGAGATCCACA), Bcl-2 (5′-GAGTACCTGAACCGGCATCT and 3′-GAAATCAAACAGAGGTCGCA), Bim (5′-TACACAAGGAGGGTGTTTGC and 3′-TCAATGCCTTCTCCATACCA) and Mcl-1 (5′-GCTTCATCGAACCATTAGCA and 3′-CCATCCCAGCCTCTTTGT).

### ATP measurement

ATP was measured using the ATP-determination kit (Molecular Probes). Cultured cells were collected and immediately placed in ice-cold ATP buffer (20 mM Tris, pH 7.5, 0.5% Nonidet P-40, 25 mM NaCl, 2.5 mM EDTA) for 5 minutes. Cell lysates were then centrifuged at 13,000 g for 30 minutes and the supernatant was measured for protein concentration.

### Oxygen consumption and lactate production

Oxygen consumption assay was performed as described [36]. Equal number of live cells were suspended at 3–5×10^6^ cells/ml in complete DMEM medium and analyzed at 37°C using a Clarke electrode. Cells were treated with oligomycin (1–2 µg/ml) to confirm that oxygen was being consumed for ATP synthesis. To measure lactate production, equal number of live cells were washed and resuspended in DMEM (5–15×10^6^ cells/ml) and aliquots were quenched at four time points over 2–3 hours using perchloric acid (0.6 M). After the removal of cellular debris and neutralization with NaOH, lactate concentrations at ≥3 time points were determined by incubating aliquots of sample (10–20 µl) with lactate dehydrogenase (1 µl) and glutamate-pyruvate transaminase (0.375 µl; Sigma Aldrich) in buffer (230–240 µl) containing glutamate (116 mM) and NAD (0.96 mM) at pH 8.9. Lactate levels were determined using a standard curve by monitoring absorption at 340 nM and the rate of lactate production was calculated as a function of time and cell concentration [37]. The ATP production from OXPHOS or glycolysis was calculated as ATP OXPHOS = 5.6×O_2_ Consumption and ATP Glycolysis = Lactate Production+0.4×O_2_ Consumption [37].

### Glucose uptake assay

Glucose uptake assay was performed as described [38].

### Measurement of ROS, mitochondria mass and mitochondrial membrane potential (ΔΨm)

The ROS, mitochondria mass and ΔΨm were measured by dihydrofluorescein diacetate (DFDA), MitoTracker Green and tetramethylrhodamine methyl ester (TMRM), respectively, as previously described [39].

### Statistical analysis

Comparison of two means was analyzed using the two-tailed unpaired Student *t* test. Values of *p* less than 0.05 were considered significant.

## Results

### Rapamycin-treated CD8^+^ T cells have enhanced ability to survive glucose withdrawal

**Figure 1:**
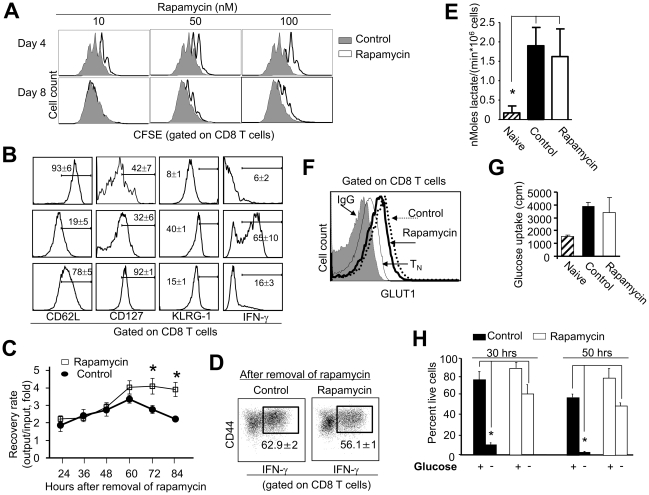
Rapamycin CD8^+^ T cells upregulate glycolysis and have enhanced ability to resist glucose withdrawal. P14 CD44^lo^CD8^+^ T_N_ were labeled with CFSE and cultured *in vitro* with B6 DCs, 10^−8^ M gp33, 5 ng/ml IL-2 and different doses of rapamycin. (A) Histograms show CFSE intensity in T cells recovered at indicated time points after culture. (B) Histograms show the expression of indicated surface markers on activated T cells that were cultured with or without rapamycin (100 nM) for 7 days. (C and D) Control and rapamycin T cells were collected from the primary culture 7 days after culture and completely washed with PBS to remove rapamycin. Equal number of live cells (0.5×10^5^ cells/well) was replated in 96-well plate in secondary culture containing IL-2 (5 ng/ml) without addition of rapamycin. Cells were collected, counted and calculated for the recovery rate (output versus input) at indicated time points after secondary culture (C). Cells collected at 72 hours after secondary culture were assessed for their production of IFN-γ. Dot plots show the fraction of cells producing IFN-γ (D). Graphs show the lactate levels in these T cells (E). Histograms show the expression of GLUT1 (F). (G) Graphs show the capability of glucose uptake. (H) Cells recovered from primary cultures were washed three times and then reseeded in the medium without glucose for additional 30 or 50 hours. At the indicated time points, cells were collected, stained with trypan blue and counted. Live cell ratio = trypan blue negative cell number/total cell number×100%. Data are shown as means ± SD and representative from five independently performed experiments. *p<0.05, significant difference.

To determine the metabolic characteristics of antigen-activated CD8^+^ T cells, we established an *in vitro* cell culture system to derive a large number of rapamycin-treated T cells. P14 CD8^+^ T_N_ were activated by B6 DCs pulsed with LCMV gp33 peptide (10^−8^ M) with or without addition of rapamycin. P14 CD8^+^ T_N_ that were cultured in the absence of rapamycin (termed control T cells) vigorusly proliferated in response to gp33 (Fig. 1A). Addition of rapamycin reduced dividing of antigen-activated CD8^+^ T cells (termed rapamycin T cells) during early time of culture (day 4, Fig. 1A). As compared to control T cells, rapamycin T cells expressed higher levels of CD62L and CD127 but lower levels of KLRG1. These rapamycin T cells produced minimal amount of IFN-γ (Fig. 1B). This phenotype of rapamycin T cells coincides with that of antigen-specific memory T cell precursors derived *in vivo* from rapamycin-treated mice [31]. Addition of rapamycin did not affect the upregulation of activation markers CD44 and CD25 on the surface of gp33-stimulated T cells (data not shown). Interestingly, when these antigen-activated T cells were replated in secondary cultures without rapamycin, we recovered significantly more live cells from the culture of rapamycin T cells than that of control T cells (Fig. 1C). Intracellular cytokine staining showed that rapamycin T cells derived from this culture produced similarly high levels of IFN-γ as did control cells (Fig. 1D), suggesting that rapamycin T cells are not anergic.

Antigen-activated T cells undergo a metabolic conversion from basal levels of OXPHOS to high rates of glycolysis [5]. We next assessed whether rapamycin treatment affected upregulation of glycolysis in antigen-activated T cells by measuring the production of lactate, expression of glucose transporter 1 (GLUT1) and uptake of glucose. As compared to T_N_, both control and rapamycin T cells produced significantly higher levels of lactate (Fig. 1E), and similarly increased the expression of GLUT1 (Fig. 1F) and uptake of glucose (Fig. 1G). These results suggest that rapamycin treatment did not impair glycolysis in antigen-activated T cells.

To assess whether glucose was critical to the survival of these activated T cells, we recovered control and rapamycin T cells 7 days after primary culture and re-seeded them in secondary cultures without glucose. Interestingly, there were significantly more (5 to 6-fold) rapamycin T cells recovered from the secondary culture than control T cells (Fig. 1H). Thus, despite their upregulated glycolysis, rapamycin T cells have enhanced ability to survive glucose withdrawal.

### Rapamycin T cells have enhanced capability of resisting growth factor withdrawal

**Figure 2:**
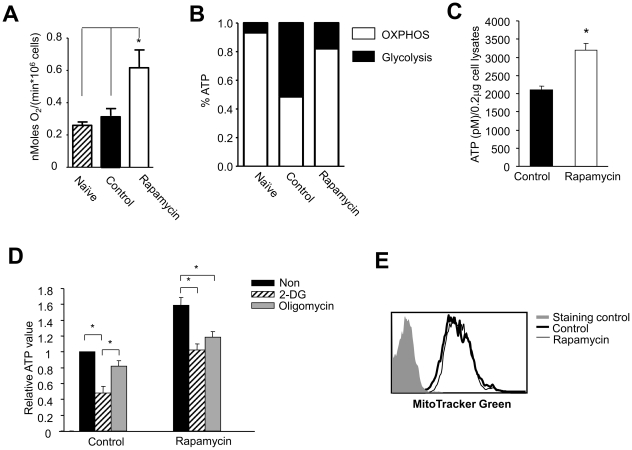
Rapamycin CD8^+^ T cells upregulate OXPHOS. P14 CD44^lo^CD8^+^ T_N_ were stimulated for 6 days *in vitro* with B6-derived DCs pulsed with gp33 plus IL-2, in the absence or presence of rapamycin (100 nM). (A) Graphs show the oxygen consumption in tested T cells. (B) The ATP production from OXPHOS or glycolysis was calculated as described in material and methods. (C) Graphs show the ATP levels in rapamycin T cells and control T cells. (D) T cells were collected from the primary culture 6 days after culture, washed three times, and re-seeded in secondary cultures treated with or without 2-DG or oligomycin. Two hours later, cells were collected for measuring the ATP levels. Graphs show the ATP levels in different T cells. (E) Histograms show the MitoTracker Green staining. Data are represented as means ± SD. Results are representative of three independently performed experiments. *p<0.05, significant difference.

It is possible that increased OXPHOS might account for enhanced ability of rapamycin T cells to survive glucose withdrawal. To test this hypothesis, we analyzed OXPHOS in antigen-activated T cells by measuring their oxygen consumption [40], [41], [42], [43]. As shown in Fig. 2A, rapamycin T cells consumed significantly more oxygen (2 to 3-fold) than control T cells. Based on these measurements (Fig. 1E and Fig. 2A), we calculated ATP production from the pathways of glycolysis and OXPHOS, respectively. Control T cells produced approximate 50% of their ATP through glycolysis, whereas rapamycin T cells produced 82% of ATP from OXPHOS (Fig. 2B).

To further determine the contribution of OXPHOS and glycolysis to the production of ATP in rapamycin T cells, we added the glycolysis inhibitor 2-deoxyglucose (2-DG) [44], [45] and mitochondrial oxidation inhibitor oligomycin [37] to the culture. We found that rapamycin T cells had significantly higher levels of ATP than control T cells at the base line (Fig. 2C). Addition of either oligomycin or 2-DG markedly reduced ATP levels in rapamycin T cells (Fig. 2D). In contrast, addition of 2-DG but not oligomycin significantly reduced ATP content in control T cells (Fig. 2D). These results suggest that rapamycin T cells were more susceptible than control cells to the inhibitory effect of oligomycin in terms of ATP production. We found that there was no difference in mitochondrial mass between rapamycin and control T cells (Fig. 2E).

**Figure 3:**
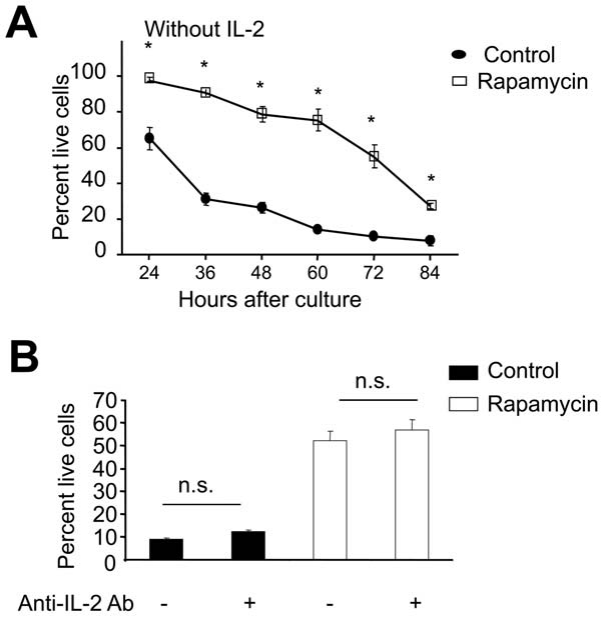
Rapamycin CD8^+^ T cells increase the ability to resist IL-2 withdrawal. P14 CD44^lo^CD8^+^ T_N_ were stimulated for 6 days *in vitro* with B6-derived DCs pulsed with gp33 plus IL-2, in the absence or presence of rapamycin (100 nM). Control CD8^+^ T cells and rapamycin CD8^+^ T cells were then collected, washed three times and seeded in secondary cultures without addition of IL-2 (A), or with addition of neutralizing anti-IL-2 Ab (B). Cells were recovered at indicated time points from secondary cultures, counted and calculated the live cell ratio as described in Fig. 1. Data are represented as means ± SD. Results are representative of three independently performed experiments. *p<0.05, significant difference. n.s., no significant difference.

Previous studies have demonstrated that OXPHOS is important for T cells to survive growth factor withdrawal [19]. To determine whether rapamycin T cells had greater ability than control T cells to resist growth factor withdrawal, we replated these T cells in secondary cultures in the absence of IL-2. As shown in Fig. 3A, the recovery rate of live control T cells was rapidly reduced to 65% by 24 hours after IL-2 starvation and further declined to 10% by 72 hours. In contrast, about 100% and 60% of rapamycin T cells were still alive by 24 and 72 hours after IL-2 withdrawal, respectively. Addition of neutralizing anti-IL-2 Ab to the culture did not affect the ability of rapamycin T cells to survive growth factor starvation (Fig. 3B). This ruled out the possibility that endogenous IL-2 could account for improved survival of rapamycin T cells. Thus, rapamycin T cells increase mitochondrial OXPHOS and have acquired the ability to resist growth factor withdrawal.

### Rapamycin T cells have augmented ability to become long-lived memory cells

**Figure 4:**
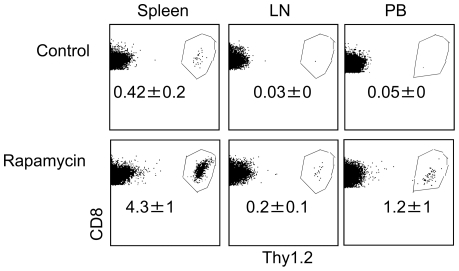
Rapamycin CD8^+^ T cells have enhanced ability to survive *in vivo*. P14 CD44^lo^CD8^+^ T_N_ were stimulated for 6 days *in vitro* with B6-derived DCs pulsed with gp33 plus IL-2, in the absence or presence of rapamycin (100 nM). T cells were collected 6 days after culture, washed and injected via tail vein into normal Thy1.1 B6 mice. One day later, donor cells were recovered from the spleen, LN and PB for measuring the number of infused donor T cells. Dot plots show the fraction of donor T cells. All data are shown as means ± SD and representative of two independently performed experiments with 3 mice in each group. *p<0.05, significant difference.

To determine the capability of rapamycin T cells to survive *in vivo*, we transplanted CFSE-labeled rapamcyin T cells to normal Thy1.1^+^ B6 mice, with CFSE-labeled control T cells transferred as controls. Twenty four hours later, there were 10- to 34-fold more P14 CD8^+^ T cells in the spleen, LN and peripheral blood (PB) of mice receiving rapamycin T cells than that of mice receiving control T cells (Fig. 4). Few donor T cells were detected in the liver, lung and BM of either rapamycin T cell recipients or control T cell recipients at this time point (data not shown), ruling out the possibility that different tissue distribution may account for the difference between these two groups. Furthermore, donor T cells had not undergone cell division by 24 hours after adoptive transfer as assessed by the intensity of CFSE (data not shown). This suggests that increased recovery of rapamycin T cells likely results from their improved ability to survive *in vivo* after adoptive transfer.

**Figure 5:**
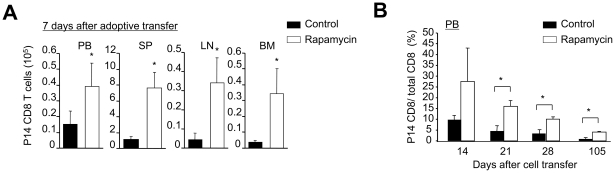
Rapamycin T cells are the precursors of long-lived memory cells. P14 CD44^lo^CD8^+^ T_N_ were stimulated *in vitro* with B6-derived DCs pulsed with gp33 plus IL-2, in the absence or presence of rapamycin (100 nM). Six days later, T cells were collected, and injected into sublethally irradiated Thy1.1 B6 mice. (A) Cells were recovered from these Thy1.1 B6 mice 7 days after transfer, counted and analyzed by flow cytometry. Graphs show the number of P14 CD8^+^ T cells in different tissues. (B) The percentage of P14 CD8^+^ T cells in the PB of these recipients was analyzed by flow cytometry at different time points as indicated. Data are shown as means ± SD and representative of two independently performed experiments with 3 mice in each group. *p<0.05, significant difference.

Previous studies have demonstrated that prior depletion of lymphocytes in the host can improve the ability of T cells to survive *in vivo* [46], [47]. To compare the ability of rapamycin T cells to generate memory T cells *in vivo* with that of control T cells, we therefore adoptively transferred rapamycin T cells (Thy1.2^+^) into sublethally irradiated lymphopenic Thy1.1^+^ B6 mice. Seven days after transfer, there were 5- to 6-fold more P14 CD8^+^ T cells recovered from mice receiving rapamycin T cells than mice receiving control T cells (Fig. 5A). This significant difference between two groups persisted throughout a period of 105 days after adoptive transfer, although both rapamycin and control T cells gradually declined in PB over time (Fig. 5B).

**Figure 6:**
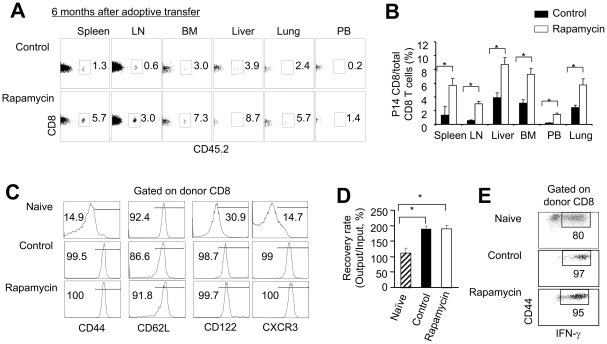
Rapamycin T cells become long-lived memory T cells *in vivo*. P14 CD44^lo^CD8^+^ T_N_ were stimulated *in vitro* with B6-derived DCs pulsed with gp33 plus IL-2, in the absence or presence of rapamycin (100 nM). Six days later, T cells were recovered, washed and adoptively transferred into sublethally irradiated Thy1.1 B6 mice. Donor T cells were collected from these Thy1.1 B6 recipient 6 months after transfer. Graphs show the percentage (A) and number (B) of P14 CD8^+^ derived CD8^+^ T cells recovered from different organs. (C) Histograms show the expression of indicated surface markers on P14 naïve T cells and donor CD8 T cells recovered from mice receiving rapamycin T cells and mice receiving control T cells. (D and E) P14 CD8^+^Thy1.2^+^ cells (1×10^4^) were highly purified using cell sorter and were stimulated *in vitro* with B6 DCs pulsed with gp33 (10^−13^ M) for another 8 days. P14 CD8^+^ naïve T cells stimulated with B6 DCs pulsed with gp33 (10^−13^ M) for 6 days were analyzed as controls. Cells were recovered from the cultures and counted. Graphs show the number of cells (D). Dot plots show the fraction of IFN-γ producing cells (E). Data are shown as means ± SD and representative of two independently performed experiments with 3 mice in each group. *p<0.05, significant difference.

Six months later, there were about 4- to 5-fold more P14 CD8^+^ T cells in both lymphoid and non-lymphoid tissues of mice receiving rapamycin T cells than that of mice receiving control T cells (Fig. 6A–B). Interestingly, donor T cells that were derived from either rapamycin T cell recipients or control T cell recipients 6 months after transfer expressed similarly high levels of CD44, CD62L, CD122 and CXCR3 (Fig. 6C). This indicates that these recovered donor T cells have a typical phenotype of long-lived memory T cells *in vivo* [15], [31], [48], [49], [50], [51], [52]. Furthermore, these donor T cells derived from both groups had similar ability to proliferate and produce high levels of IFN-γ (Fig. 6D–E). Thus, there was no difference in the phenotype and function at the single cell level between memory cells derived from rapamycin T cells and memory cells derived from control T cells. Altogether, these data indicate that rapamycin T cells may contain higher frequency of memory precursors that are able to become long-lived memory T cells *in vivo* than control T cells.

### OXPHOS is critical to the survival of rapamycin T cells

**Figure 7:**
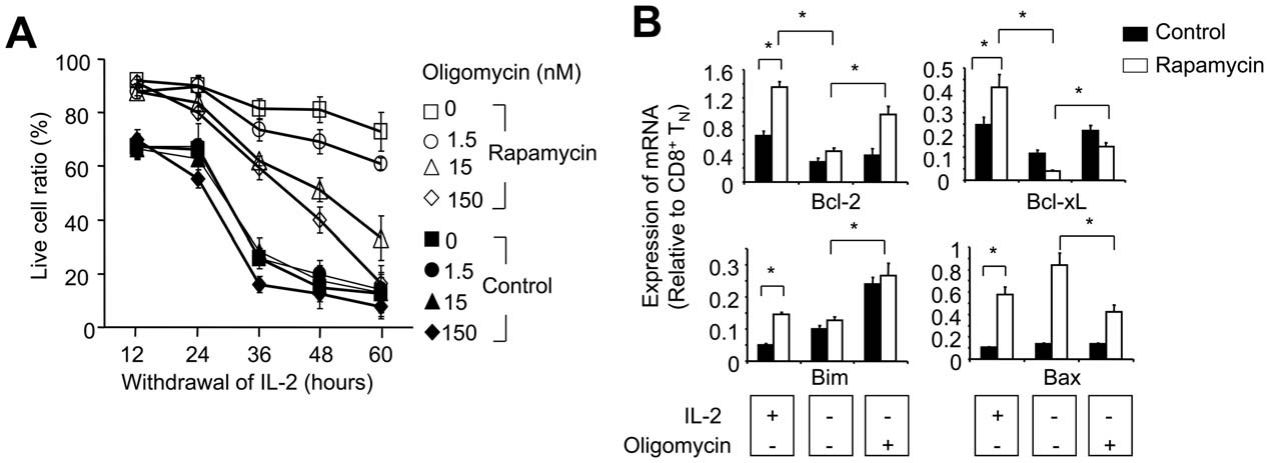
Inhibition of OXPHOS in rapamycin T cells by oligomycin reduces their survival capability of resisting IL-2 withdrawal. P14 CD44^lo^CD8^+^ T_N_ were stimulated *in vitro* with B6-derived DCs pulsed with gp33 plus IL-2, in the absence or presence of rapamycin (100 nM). (A) Six days later, cells were collected, and washed three times and seeded in secondary cultures in the absence IL-2, with or without addition of oligomycin. Cells were collected at the indicated time points, counted and calculated the live cell ratio as described in Fig. 1. (B) Control and rapamycin CD8^+^ cells recovered from cultured were re-seeded for the secondary culture in the presence of IL-2, or in the absence of IL-2 with or without addition of oligomycin for 24 hours. Quantitative real-time RT-PCR was performed to measure the expression of Bcl-2, Bcl-xL, Bim and Bax. Data are shown as means ± SD and representative of three independently performed experiments. *p<0.05, significant difference.

To further determine the importance of OXPHOS in rapamycin T cells, we used oligomycin to block the final step of OXPHOS. As shown in Fig. 7A, oligomycin dose-dependently reduced the recovery of live rapamycin T cells cultured without IL-2. In contrast, control T cells rapidly diminished in the same culture condition even in the absence of oligomycin. These data suggest that rapamycin T cells are sensitive to oligmycin-mediated inhibition of OXPHOS, whereas control T cells may die through a mechanism independent of oligomycin treatment.

Oligomycin could kill rapamycin T cells by inducing apoptosis and/or metabolic collapse. To distinguish between these two possibilities, we first examined the expression of genes associated with pro- and anti-apoptosis. Real-time RT-PCR analysis showed that rapamycin T cells expressed higher levels of Bcl-2, Bcl-xL, Bax and Bim than control T cells (Fig. 7B). Withdrawal of IL-2 significantly reduced the expression of Bcl-2 and Bcl-xL in both rapamycin and control T cells, but had no effect on their expression of Bax and Bim (Fig. 7B). However, this reduced expression of anti-apoptotic genes Bcl-2 and Bcl-xL did not support the improved survival of rapamycin T cells in the absence of IL-2 (Fig. 3A). Furthermore, addition of oligomycin resulted in a significant upregulation of both Bcl-2 and Bcl-xL in rapamycin T cells as compared to oligomycin-untreated counterparts (Fig. 7B). This could not explain why oligomycin reduced survival of rapamycin T cells. Thus, although increased expression anti-apoptotic genes (e.g. Bcl-2 and Bcl-xL) could be associated with improved survival of rapamycin-treated T cells [26], [27], [28], [29], [30], it is possible that oligomycin-mediated killing of rapamycin T cells may be not due to decreased levels of anti-apoptotic Bcl-2 or Bcl-xL.

**Figure 8:**
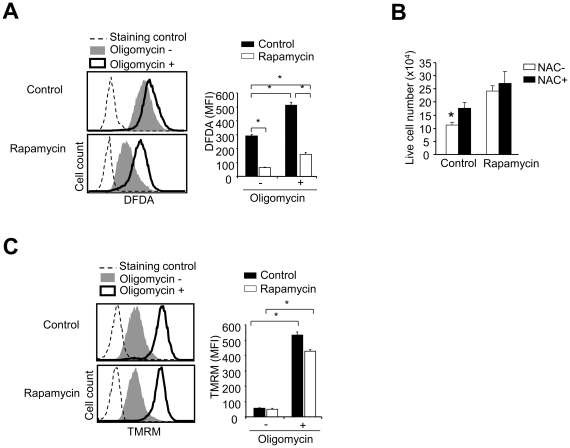
Inhibition of OXPHOS rapamycin T cells by oligomycin induces high levels of ROS and hyperpolarization of mitochondrial membrane. P14 CD44^lo^CD8^+^ T_N_ were stimulated *in vitro* with B6-derived DCs pulsed with gp33 plus IL-2, in the absence or presence of rapamycin (100 nM). Six days later, cells were collected, and washed three times and seeded in secondary cultures in the absence IL-2, with or without addition of oligomycin, or with or without addition of NAC. (A) Cells were collected 2 hours after culture. Histograms show the ROS levels measured by flow cytometry and the graphs show the mean fluorescence intensity (MFI) of ROS. (B) Graphs show the live cell number of T cells in the secondary culture 24 hours after culture in the absence of IL-2 with or without NAC treatment. (C) Histograms show ΔΨm determined by flow cytometry and the graphs show the MFI. Data are shown as means ± SD and representative of three independently performed experiments. *p<0.05, significant difference.

It has been demonstrated that increased ROS, mitochondrial hyperpolarization and ATP depletion represent early steps of T cell apoptosis [53]. Flow cytometry analysis showed that rapamycin T cells had lower levels of ROS than control T cells (Fig. 8A). We then asked whether antioxidants protected control T cells from ROS-mediated cell death. Rapamycin and control T cells were replated in secondary cultures in the absence of IL-2, with or without addition of antioxidant N-acetylcysteine (NAC) for 24 hours. As shown in Fig. 8B, control T cells had 30% more live cells in the culture treated with NAC than the culture without NAC treatment. In contrast, NAC treatment had no impact on the recovery of live cells in the culture of rapamycin T cells. These results suggest that increased ROS in control T cells may account, at least in part, for their impaired ability to resist growth factor withdrawal. Interestingly, treatment with oligomycin increased the levels of ROS in both rapamycin and control T cells (Fig. 8A). Furthermore, oligomycin increased mitochondrial membrane potential (ΔΨm) in both rapamycin and control T cells (Fig. 8C). Thus, it is likely that inhibition of OXPHOS by oligomycin may result in a metabolic collapse in rapamycin T cells.

## Discussion

Antigen-activated T cells undergo metabolic conversion from basal levels of OXPHOS to high rates of glycolysis to meet their bioenergetic and biosynthetic demand [5], [6]. We demonstrate here that antigenic-stimulation of CD8^+^ T cells in the presence of rapamycin increases both OXPHOS and glycolysis, and augments the formation of memory cell precursors and their long lived memory cell progenies. These rapamycin CD8^+^ T cells resisted both glucose and IL-2 withdrawal *in vitro* and had augmented capability to survive *in vivo*. Inhibition of OXPHOS by oligomycin in rapamycin T cells resulted in their ATP reduction, mitochondrial hyperpolarization, increased ROS levels and decreased ability to survive growth factor withdrawal. Our findings are consistent with previous observations that oxidative metabolism is critical to the persistence of chronically activated T cells in diseases such as rheumatoid arthritis, psoriasis, lupus and graft-versus-host disease [36], [54], [55], [56], [57] and that administration of rapamycin enhances the *in vivo* generation of memory precursor cells [31].

Enhanced generation of memory T cells relies on improved survival of memory cell precursors [14], [17], [19], [31], [52], [58], [59]. Several lines of evidence indicate that augmented oxidative metabolism may play an essential role in improving the survival of rapamycin CD8^+^ T cells. For example, deletion of Traf6, which encodes a signal regulating fatty acid oxidation, impairs the transition of effector T cells to memory cells [19]. *In vivo* administration of metformin, a metabolic regulator of fatty acid oxidation [4], increases the survival of antigen-activated T cells and their differentiation into memory T cells [19]. These observations suggest that OXPHOS is important for the formation of memory T cells. We found that rapamycin CD8^+^ memory cell precursors significantly increased the rate of OXPHOS and acquired an augmented ability to survive in cultures in the absence of either glucose or growth factors. Furthermore, inhibiting OXPHOS with oligomycin abrogated the ability of rapamycin CD8^+^ T cells to survive growth factor withdrawal. Thus, further investigating the mechanisms by which increased oxidation metabolism improves the survival of antigen-activated T cells will be important for augmenting the generation of memory T cells.

Although previous studies suggest that upregulation of the anti-apoptotic genes Bcl2 and Bcl-xL might be important to improving the survival of rapamycin-treated T cells [26], [27], [28], [29], [30], we confirmed that rapamycin T cells expressed higher levels of Bcl-2 and Bcl-xL than control T cells in cultured supplemented with IL-2. This may explain our results that rapamycin T cells had greater ability than control T cells to survive in the presence of growth factor. However, both rapamycin T cells and control T cells down-regulated their expression of Bcl-2 and Bcl-xL to the same low level in response to IL-2 withdrawal, rapamycin T cells still had greater ability than control T cells to survive under this condition. This suggests that enhanced survival of rapamycin T cells in the absence of IL-2 cannot be simply explained by the expression of Bcl-2 and Bcl-xL. Interestingly, we found that oligomycin inhibition of OXPHOS led to a bioenergetic collapse in rapamycin T cells in the culture without IL-2 supplement, such as mitochondrial membrane hyperpolarization, increased ROS and reduced ATP levels. Surprisingly, oligomycin increased the expression of Bcl-2 and Bcl-xL in IL-2 deprived rapamycin T cells. These data suggest that enhanced OXPHOS in rapamycin T cells may be critical for preventing them from bioenergetic collapse in response to growth factor withdrawal, thereby improving their survival capability.

In addition to increased OXPHOS, upregulated glycolysis may be also important for enhanced survival capability of rapamycin T cells. We observed that treatment of rapamycin T cells with 2-DG resulted in a reduction of ATP levels in rapamycin to that treated with oligomycin. This suggests that upregulated glycolysis may also play an important role in rapamycin T cells, which can be explained in several aspects. First, since 2-DG may also reduce the production of pyruvate, a glucose-derived metabolite that is oxidized in mitochondria through the tricarboxylic cycle [44], [45], it is possible that 2-DG-mediated reduction of ATP in rapamycin T cells might be partially accounted for by a decrease in OXPHOS of glucose-derived metabolites. Second, in addition to producing ATP, high rates of glycolysis help maintain antioxidant levels in activated T cells (e.g. pyruvate and glutathione), which can prevent ROS from being formed or remove them before they damage the cell [4], [5]. This might explain how increased oxidative metabolism in rapamycin T cells did not result in enhanced levels of ROS, which could cause DNA damage and cell death [60], [61], [62], [63], [64], [65], [66]. Thus, it is possible that the hybrid phenotype of increased glycolysis and OXPHOS in rapamycin T cells could provide an optimized cellular metabolism to support their survival and generation of memory cell precursors.

A better understanding of how rapamycin treatment creates this hybrid metabolic phenotype in memory T cell precursors may lead to the development of novel approaches to augment the formation and maintenance of long-lived memory cells. Several studies suggest that rapamycin treatment increases fatty acid oxidation while reducing glycolysis in muscle cells [24], whereas others suggest that rapamycin decreases oxidative metabolism in leukemic cell line Jurkat cells. These data suggest that rapamycin regulation of cellular metabolism may be context dependent due to the nature of other available signals. Although mTOR is critical for TCR/CD28-driven T cell proliferation and upregulation of glycolysis [2], [25], we found that inhibition of mTOR by rapamycin neither prevented the upregulation of activation markers in antigen-stimulated T cells nor reduced the expression of GLUT-1 and glucose uptake. It has been demonstrated that Pim kinases are required for T cell growth and proliferation in the presence of rapamycin [3]. Whether Pim kinases may be critical to the regulation of cellular metabolism in rapamycin T cells has yet to be determined.

It will be interesting to determine whether rapamycin treatment increases the frequency of memory T cells or generates a cell with distinctive metabolic properties from a rapamycin-untreated cell. Many studies have demonstrated that antigen-activated CD62L^hi^ T cells (central memory cells) but not CD62L^lo^ T cells (effector and effector memory cells) are responsible for the generation of long-lived memory T cells [15], [31], [48], [49], [50], [51], [52], [67]. CD62L^lo^ T cells derived from *in vitro* cultures may rapidly diminish *in vivo* following adoptive transfer [12], [13], [68], [69]. We found that rapamycin-treated T cells contained 4-fold more CD62L^hi^ T cells in frequency than control T cells, whereas control T cells had more CD62L^lo^ T cells in fraction than rapamycin-treated T cells. This coincides with our observation that there were 4- to 5-fold more long-lived memory T cells in mice receiving rapamycin-treated T cells than mice receiving control T cells. Furthermore, we observed that at the single cell level these long-lived memory T cells derived *in vivo* from either rapamycin-treated T cells or control T cells expressed the same phenotype and had similar ability to proliferate and produce IFN-γ. These rapamycin-treated T cells acquired effector traits in response to antigenic stimulation. Thus, it is likely that rapamycin treatment may increase the frequency of antigen-specific memory T cells.

However, we do not rule out the possibility that CD62L^hi^ and CD62L^lo^ T cells derived from rapamycin-treated cultures could have different metabolic properties from their counterparts generated in the absence of rapamycin. It has been demonstrated that CD62L^lo^ effector T cells isolated from rapamycin-treated mice have greater ability than CD62L^lo^ effector T cells derived from rapamycin-untreated mice to generate more functional memory T cells [31]. Most importantly, these rapamycin-treated CD62L^lo^ effector T cells have enhanced capability of undergoing conversion to CD62L^hi^ central memory cells *in vivo* after adoptive transfer [31]. These data suggest that rapamycin treatment could enhance the plasticity of CD62L^lo^ effector T cells to develop into central memory T cells. In our present study, the metabolic properties of rapamycin-treated T cells could mainly reflect the features of CD62L^hi^ T cells, and that of control T cells could be accounted for by CD62L^lo^ T cells. Thus, future studies should be investigating whether the metabolic features of rapamycin-treated T cells can be acquired by memory T cells in the absence of rapamycin and whether rapamycin-treated T cells may change their metabolic features during immune response.

In summary, our data show that treatment of antigen-activated CD8^+^ T cells by rapamycin upregulated both OXPHOS and glycolysis. This hybrid metabolism phenotype in rapamycin CD8^+^ T cells correlates with their enhanced ability to survive growth factor withdrawal and augmented generation of memory T cells. Thus, *in vitro* generated rapamycin T cells may represent a unique cell model for identifying nutrients and metabolism-regulating signals critical to memory cell precursors, thereby improving the formation and maintenance of memory T cells.
